# Olfactory and Gustatory Dysfunction in Patients With Autoimmune Encephalitis

**DOI:** 10.3389/fneur.2019.00480

**Published:** 2019-05-14

**Authors:** Rohat Geran, Florian C. Uecker, Harald Prüss, Karl Georg Haeusler, Friedemann Paul, Klemens Ruprecht, Lutz Harms, Felix A. Schmidt

**Affiliations:** ^1^Department of Neurology, Clinical and Experimental Multiple Sclerosis Research Center, Charité-Universitätsmedizin Berlin, Corporate Member of Freie Universität Berlin, Humboldt-Universität zu Berlin, and Berlin Institute of Health, Berlin, Germany; ^2^Center for Stroke Research, Charité-Universitätsmedizin Berlin, Corporate Member of Freie Universität Berlin, Humboldt-Universität zu Berlin, and Berlin Institute of Health, Berlin, Germany; ^3^Department of Neurology, Charité-Universitätsmedizin Berlin, Corporate Member of Freie Universität Berlin, Humboldt-Universität zu Berlin, and Berlin Institute of Health, Berlin, Germany; ^4^Department of Otorhinolaryngology, Head and Neck Surgery, Charité-Universitätsmedizin Berlin, Corporate Member of Freie Universität Berlin, Humboldt-Universität zu Berlin, and Berlin Institute of Health, Berlin, Germany; ^5^German Center for Neurodegenerative Diseases (DZNE), Berlin, Germany; ^6^Department of Neurology, Universitätsklinikum Würzburg, Würzburg, Germany; ^7^NeuroCure Clinical Research Center, Charité-Universitätsmedizin Berlin, Corporate Member of Freie Universität Berlin, Humboldt-Universität zu Berlin and Berlin Institute of Health, Berlin, Germany; ^8^Experimental and Clinical Research Center, Max Delbrueck Center for Molecular Medicine and Charité-Universitätsmedizin Berlin, Corporate Member of Freie Universität Berlin, Humboldt-Universität zu Berlin and Berlin Institute of Health, Berlin, Germany; ^9^Berlin Institute of Health, Berlin, Germany

**Keywords:** autoimmune encephalitis, olfactory dysfunction, gustatory dysfunction, olfactory testing, threshold discrimination identification test

## Abstract

**Objective:** To test the hypothesis that olfactory (OF) and gustatory function (GF) is disturbed in patients with autoimmune encephalitides (AE).

**Methods:** The orthonasal OF was tested in 32 patients with AE and 32 age- and sex-matched healthy controls (HC) with the standardized Threshold Discrimination Identification (TDI) score. This validated olfactory testing method yields individual scores for olfactory threshold (T), odor discrimination (D), and identification (I), along with a composite TDI score. The GF was determined by the Taste Strip Test (TST).

**Results:** Overall, 24/32 (75%) of patients with AE, but none of 32 HC (*p* < 0.001) had olfactory dysfunction in TDI testing. The results of the threshold, discrimination and identification subtests were significantly reduced in patients with AE compared to HC (all *p* < 0.001). Assessed by TST, 5/19 (26.3%) of patients with AE, but none of 19 HC presented a significant limitation in GF (*p* < 0.001). The TDI score was correlated with the subjective estimation of the olfactory capacity on a visual analog scale (VAS; r_s_ = 0.475, *p* = 0.008). Neither age, sex, modified Rankin Scale nor disease duration were associated with the composite TDI score.

**Conclusions:** This is the first study investigating OF and GF in AE patients. According to unblinded assessment, patients with AE have a reduced olfactory and gustatory capacity compared to HC, suggesting that olfactory and gustatory dysfunction are hitherto unrecognized symptoms in AE. Further studies with larger number of AE patients would be of interest to verify our results.

## Introduction

In recent years, the term autoimmune encephalitis was established for a heterogeneous group of antibody-associated disorders of the brain that can either be caused by paraneoplastic or non-paraneoplastic conditions (1, 2). AE is characterized by a subacute onset of working memory deficits, psychiatric symptoms, and altered mental status. According to international consensus, the diagnosis of definite autoimmune limbic encephalitis can be made, if the following criteria are met: MRI abnormalities of the medial temporal lobe, epileptic slow-waves on EEG, and CSF pleocytosis (3). In anti-NMDA receptor encephalitis fewer than half of MRIs reveal abnormal findings (4, 5). Presence of antibodies in CSF and serum facilitate the diagnosis as well as a positive treatment response to immunotherapy (6, 7). In 50–60% of AE patients oligoclonal bands in CSF are detected. Hence, AE is in particular defined by means of antibodies, which either target intracellular antigens or surface antigens especially in the limbic system. The existing diagnostics yield no definitive evidence. So further diagnostic tests are desirable. Olfactory dysfunction is an increasingly detected symptom in neuroimmunological disorders such as multiple sclerosis and neuromyelitis optica spectrum disorder (8, 9). Essential parts of the olfactory pathway are located in the limbic system. Olfactory information from the olfactory cortex (including the piriform and enthorinal cortex, the olfactory tubercle and the anterior olfactory nucleus) is projected to the hippocampus, amygdala, nucleus accumbens, and hypothalamus (10). As the olfactory information is processed in these brain areas, we hypothesized that functional disturbances of the limbic system in AE patients could lead to olfactory and gustatory dysfunction (11, 12). The aim of the study was to investigate olfactory and gustatory function in AE patients. Besides possible social, emotional and behavioral consequences of olfactory, and gustatory dysfunction, its detection using a standardized test could be a helpful marker of disease activity (13).

## Methods

### Study Participants

In this prospective case-control study, 32 patients with AE (44% women, 18–75 years, mean ± standard deviation Ø 52 ± 18 years) were examined from April 2015 to May 2016. The median (±SD) disease duration was 18 ± 13 months. In detail, 26 seropositive (81.3%) and 6 seronegative patients were recruited at in- and outpatient clinics of the Charité-Universitätsmedizin Berlin, Germany. Antibody testing was performed in all patients with seropositive and seronegative AE with the same commercially available cell-based assay (Euroimmun, Lübeck, Germany). Six AE patients with anti-leucine-rich glioma-inactivated 1 (anti-LGI1) antibodies, four with anti-N-methyl-D-aspartate receptor (anti-NMDAR) antibodies, four with antiglutamate decarboxylase (anti-GAD) antibodies, two with anti-contactin-associated protein 2 (anti-Caspr2) antibodies, two with anti-Hu-antibodies, one with anti-α-amino-3-hydroxy-5-methyl-4-isoxazolepropionic acid receptor (anti-AMPAR) antibodies, one with anti-γ-aminobutyric acid B receptor (anti-GABA-B-R) antibodies, one with anti-voltage-gated calcium channel (anti-VGCC) antibodies, one with anti-dipeptidyl-peptidase-like protein-6 (anti-DPXX) antibodies, one with anticollapsin response-mediator protein 5 (anti-CV2) antibodies, one with anti-metabotropic glutamate receptor 5 (anti-mGluR5) antibodies, and two with not characterized anti-neuronal antibodies were included in the study. The diagnosis of AE was verified by two experienced neurologists according to current peer-reviewed diagnostic criteria (3). At baseline all patients were already diagnosed and followed up, none was a de novo patient. Beyond that, none of the patients have had a herpes simplex encephalitis (HSE) in their medical history. At the time of testing eight patients received rituximab for immunotherapy, four patients were treated with plasmapheresis (between three and ten courses), four with intravenous immunoglobulins, three with steroids, and one with bortezomib (7, 14, 15). In addition, four AE patients were treated with a two-stage treatment. Three of these patients were first treated with plasmapheresis and afterwards with rituximab, and one patient was treated with immunoadsorption and afterwards with rituximab. Overall, 8 out of 32 AE patients had neither immunosuppressive therapy nor plasmapheresis at the time of OF and GF testing. The age- and sex-matched HC group comprised 32 individuals and were recruited among hospital staff.

### Inclusion- and Exclusion Criteria

The patients were included in, respectively, excluded from the study after ENT and neurological examination as well as by completing two questionnaires. Patients with the diagnosis AE were included in the study. Exclusion criteria for study participants of both groups were known olfactory disorders (caused by e.g., infections of the upper respiratory tract, post-traumatic, sinunasal, post-infectious, allergies), a major depression in medical history, age over 75 as well as pregnancy and lactation, respectively (16). To exclude olfactory disorders of sinunasal origin an endoscopic ENT examination of the nasal passage, the sinuses, and the nasal mucosa was performed in patients with AE. Furthermore, patients suffering from diseases associated with olfactory dysfunction such as Parkinson's or Alzheimer's disease, multiple sclerosis and neuromyelitis optica spectrum disorder as well as patients taking drugs that can cause olfactory dysfunction (such as amitryptilin, methotrexat, D-Penicillamine, and certain other antibiotics, e.g., aminoglycosides, macrolides, and tetracyclines) were excluded.

The Mini Mental State Examination (MMSE) was performed before OF and GF testing to exclude severe cognitive dysfunction (17). AE patients scoring at least 25 out of 30 points were included. To identify a major depression the Beck Depression Inventory-II (BDI-II) was applied (18). The BDI-II comprises 21 questions in a self-reported multiple choice form varying in severity and symptoms of depression such as guilt, hopelessness and physical symptoms such as fatigue, lack of interest in sex and weight loss. We predefined a BDI-II score higher as 15 points as exclusion criterion. Physical disability was rated according to the modified Rankin Scale (mRS) score ranging from 0 (no symptoms) to 6 (death) (19). Patients with a mRS score >3 were excluded from the study.

### Orthonasal Olfactory Function

The orthonasal OF was examined by an unblinded investigator using the tripartite TDI score (Sniffin' Sticks test battery; Burghart GmbH, Wedel, Germany), which is composed of threshold, discrimination and odor identification subtests and is recommended by the German Society for Otorhinolaryngology, Head and Neck Surgery (20). The standardized and reliable (r_tt_ = 0.72) test is designed as an alternative forced choice (AFC) test (21). The olfactory perception threshold was determined by a 16-stage dilution series of n-butanol with 48 Sniffin' Sticks. Using the AFC principle, blindfolded subjects had to identify the sniffin stick that contained the odorant. The discrimination test was performed with 48 Sniffin' Sticks of different smell qualities to test the distinction of smells. Everyday odors had to be recognized with the identification test which consist of 16 Sniffin' Sticks. A TDI score of < 16 out of 48 indicates functional anosmia, a score up to 30.5 indicates hyposmia and a score above 30.5 indicates normosmia.

### Gustatory Function

To determine the GF, the Taste Strip Test (TST; Burghart GmbH, Wedel, Germany) was applied (22). The multiple forced-choice test evaluates the qualities salty, sweet, sour and bitter, each presented in four different concentrations. A maximum score of 16 points can be achieved. A test score of below nine indicates an impaired GF.

### Secondary Outcomes

To detect confounding factors primary outcomes TDI and TST scores were tested in a linear regression model with the mRS-, BDI-, MMSE score, subjective estimation of the olfactory capacity on visual analog scale (VAS), age, disease duration and antibody status.

### Statistical Analysis

Statistical analysis was performed using SPSS 25.0 (IBM SPSS Statistics for Windows, Version 25.0. Armonk, NY: IBM Corp.). Figures were created with GraphPad Prism 7.03 (GraphPad Software Inc., La Jola, USA). The data are presented in means with standard deviations (SD) (mean ± SD). To test the normality the Shapiro-Wilk test was performed. Taking into consideration the non-Gaussian distribution and sample size, the non-parametric Wilcoxon signed-rank test for paired data was used to compare TDI and I score of AE patients with the age- and sex-matched HC. Whereas, the parametric equivalent Student's *t*-test for paired samples was applied to compare the obtained scores of the TST score as well as the T and D subtests of AE patients with HC. The AE patients were categorized into four groups (patients with antibodies against synaptic receptors, with antibodies against intracellular antigens, with antibodies against ion channels and other cell-surface proteins and without antibodies) to analyze the influence of antibodies on TDI score with the Kruskal-Wallis H test and on TST score with one-way ANOVA. Finally, ANCOVA was used to verify the absence of gender influence on TDI score. Correlations were calculated applying the Pearson and Spearman correlation. A *p-*value of < 0.05 was defined as significant.

## Results

The demographics and clinical characteristics of 32 AE patients and 32 HC examined in this study are presented in Table 1. In psychophysical TDI testing, a significant olfactory dysfunction was present in 24/32 (75%) of AE patients, but in none of the HC (*p* < 0.001). The GF was significantly limited in 26.3% (5/19) of AE patients in the TST and none of the HC (*p* < 0.001). Sixty percent of the patients with a gustatory dysfunction were also hyposmic, 20% anosmic. The OF results correlated with the subjective estimation of olfactory capacity on VAS (*r* = 0.475, *p* = 0.008).

**Table 1:** Demographic and clinical data of AE patients and HC.

**Characteristics**	**AE patients (*n* = 32)**	**HC (*n* = 32)**
**Gender**		
Female (%)	14 (44)	14 (44)
Male (%)	18 (56)	18 (56)
Age, mean (SD), y	52.5 (17.8)	52.0 (17.5)
**Time after disease onset, median (IQR), month**	19.3 (10.3–25.0)	n.a.
Tumors present (%)	4 (12.5)	0
mRS score, mean (SD)	1.7 (1.1)	0
MMSE score, mean (SD)	27.8 (1.7)	n.a.
BDI-II score, mean (SD)	8.0 (7.1)	n.a.
**Acute therapy (%)**	15 (47)	0
IV corticosteroids (%)	3 (9)	0
Plasma exchange (%)	8 (25)	0
Immunoglobulins (%)	4 (13)	0
**Long-term immunosuppression (%)**		
Oral corticosteroids (%)	7 (22)	0
Rituximab (%)	11 (34)	0
Bortezomib (%)	1 (3)	0
**Antibodies against intracellular antigens (%)**	7 (22)	0
Hu (%)	2 (6)	0
CV2 (%)	1 (3)	0
GAD (%)	4 (13)	0
**Antibodies against synaptic receptors (%)**	7 (22)	0
NMDA receptor (%)	4 (13)	0
AMPA receptor (%)	1 (3)	0
GABA_B_ receptor (%)	1 (3)	0
mGluR5 (%)	1 (3)	0
**Antibodies against ion channels and other cell-surface proteins (%)**	12 (38)	0
LGI1 (%)	6 (19)	0
CASPR2 (%)	2 (6)	0
VGCC (%)	1 (3)	0
DPPX (%)	1 (3)	0
Not characterized (%)	2 (6)	0
Oligoclonal bands (%)	6 (19)	0

*AE, autoimmune encephalitis; mRS, modified Rankin Scale score; MMSE, Mini-Mental State Examination; BDI, Beck Depression Inventory; GAD, glutamic acid decarboxylase; NMDA, N-Methyl-D-aspartate; AMPA, α-amino-3-hydroxy-5-methyl-4-isoxazolepropionic acid; GABA, gamma-aminobutyric acid; mGluR5, metabotropic glutamate receptor 5; LGI1, leucine-rich, glioma inactivated 1; CASPR2, contactin associated protein receptor 2; VGCC, voltage-gated calcium channel; DPPX, dipeptidyl aminopeptidase-like protein 6; n.a., not available*.

### Orthonasal Olfactory Function

**Figure 1:**
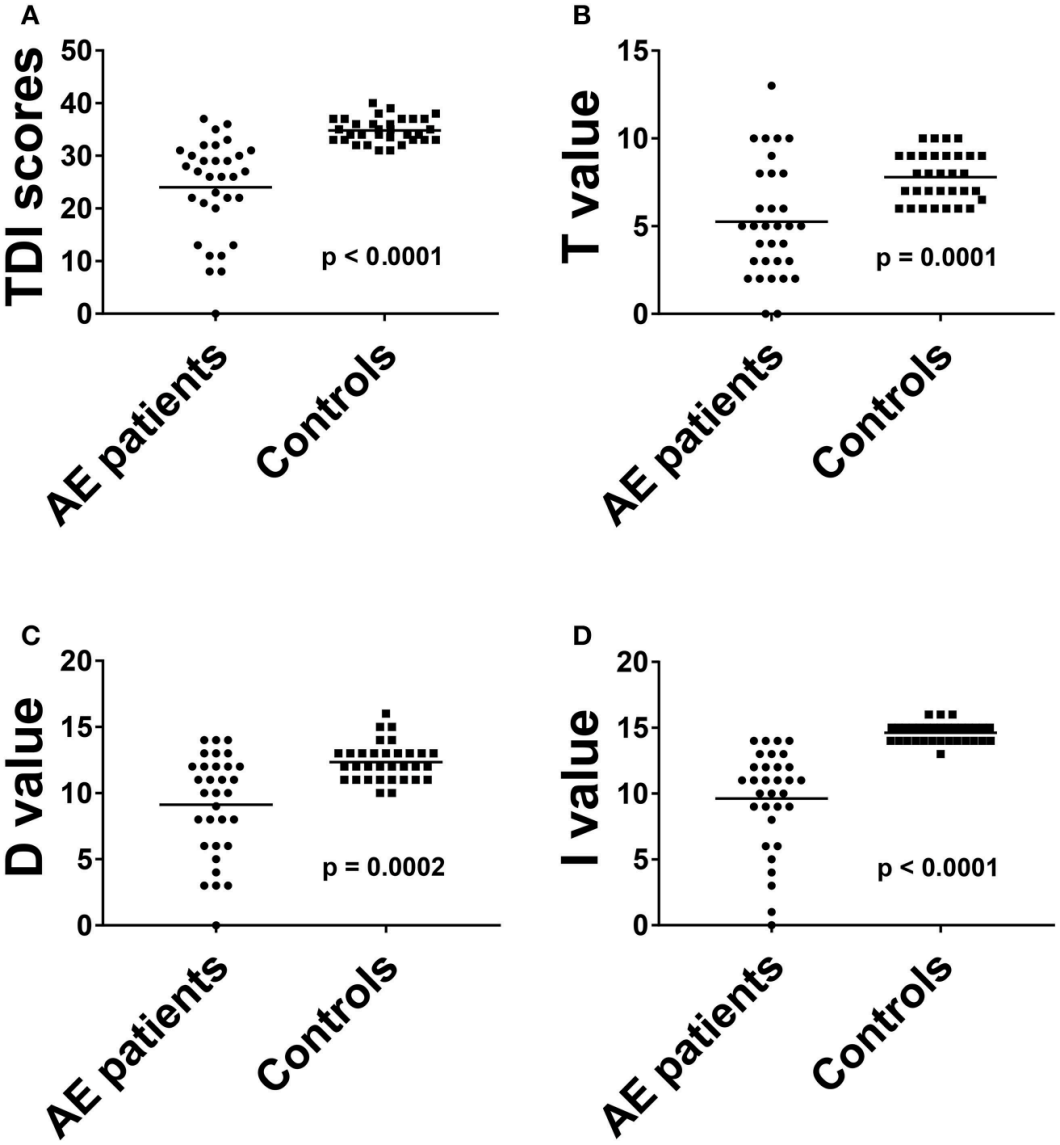
The results of total threshold, discrimination and identification scores **(A)** and of all olfactory subtests **(B–D)** in AE patients were compared to HC. Horizontal bars: mean. AE, autoimmune encephalitis; D, discrimination score; I, identification score; T, threshold score.

The overall TDI score in all 32 AE patients was 24.0 ± 9.2, the 32 HC had a median TDI score of 34.8 ± 2.3 [*p* < 0.001, (Figure 1A)]. Effect size for detecting olfactory dysfunction was *r* = 0.87. In 53% of all AE patients (17/32) hyposmia was detected with a score of 25.7 ± 3.3. 7/32 (22%) of AE patients showed anosmia with a score of 9.1 ± 4.2. All HC showed normosmia in TDI testing. Moreover, the results of the Threshold subtest (*p* < 0.001, Cohen's *d* 1.15), the Discrimination subtest (*p* < 0.001, Cohen's *d* 1.39) and the Identification subtest (*p* < 0.001, *r* = 0.85) revealed significant differences between AE patients and HC (Figures 1B–D). The specificity and the positive predictive value were 97% with a cut-off score of 13 out of 16 in the Identification subtest. In addition, the sensitivity was 88% and the negative predictive value 89%. Comparing TDI scores of female (25.9 ± 6.7) and male (22.5 ± 10.3) AE patients no significant difference was found (*p* = 0.424).

### Gustatory Functiozn

**Figure 2:**
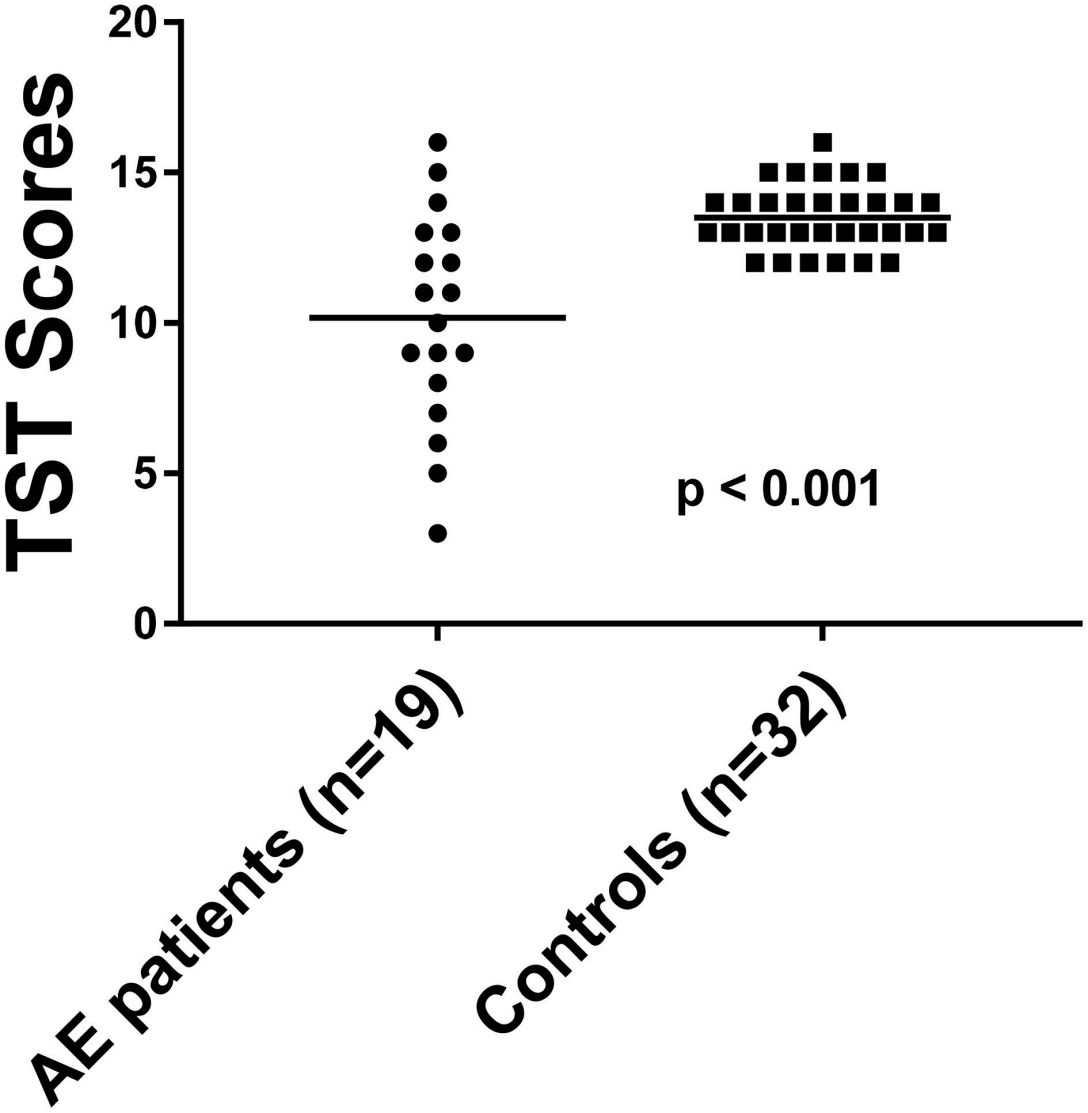
The TST score in AE patients compared to HC. Horizontal bars: mean. TST, taste stripe test.

The TST score in 19 patients was 11.2 ± 3.4 (Table 2). The TST score in the 32 HC was 13.5 ± 1.1. 26.3% of the AE patients (5/19) had reduced GF with a score of 5.8 ± 1.7, while none of the HC showed gustatory dysfunction. The GF was significantly reduced in AE patients [*p* < 0.001, (Figure 2)], and effect size for detecting gustatory dysfunction was Cohen's *d* = 1.13.

**Table 2:** Results of orthonasal and gustatory testing of AE patients and healthy controls.

	**AE patients**	**Controls**	***P*-value**
TDI score, mean (SD)	24.0 (9.2)	34.8 (2.3)	< 0.001
Hyposmia (%)	17 (53)	0	
Hyposmia, mean (SD)	25.7 (3.3)	–	
Anosmia (%)	7 (22)	0	
Anosmia, mean (SD)	9.1 (4.2)	–	
Threshold, mean (SD)	5.3 (3.2)	7.8 (1.4)	< 0.001
Discrimination, mean (SD)	9.1 (3.8)	12.3 (1.4)	< 0.001
Identification, mean (SD)	9.6 (3.8)	14.6 (0.7)	< 0.001
TST score, mean (SD)	10.2 (3.4)	13.5 (1.1)	< 0.001
Hypogeusia (%)	5/19 (26)	0/32 (0)	
Hypogeusia, mean (SD)	5.8 (1.7)	–	

*AE, autoimmune encephalitis; TDI, threshold discrimination identification test; TST, taste stripe test*.

### Secondary Outcomes

VAS was used as an instrument for subjective evaluation of OF and GF. The TDI score correlated significantly with olfactory capacity expressed on VAS. Besides quantitative self-evaluation AE patients were also asked for qualitative dysosmia. Two AE patients reported phantosmia (6.25%), none reported parosmia. The autoantibody type had no significant influence on TDI score (Chi-Square = 1.531, *p* = 0.675, *r* = 0.41) in the Kruskal-Wallis H test and in the one-way ANOVA on TST score [*F*_(3, 15)_ = 0.707, *p* = 0.563, η^2^ = 0.12]. In a linear regression model confounding factors such as MMSE, mRS, BDI, age, or disease duration had no influence on OF or GF, respectively.

## Discussion

To the best of our knowledge, this is the first study investigating OF and GF in AE patients. The majority of AE patients, but none of HC showed olfactory dysfunction. A significant loss of olfactory capacity in AE patients compared to HC was seen in all olfactory subtests measuring olfactory threshold, discrimination, and identification of smells. The threshold subtest reflects a more peripheral olfactory function, whereas discrimination and identification depict higher-level processing of olfactory information (11, 23, 24). The results of the present single-center study indicate olfactory impairment in AE patients in peripheral as well as in central olfactory processing regions.

The primary olfactory cortex such as the piriform cortex, amygdala, entorhinal cortex, and anterior olfactory nucleus receive olfactory information from the olfactory bulb and project them to regions of the secondary olfactory cortex (e.g., hippocampus, parahippocampal gyrus, insula, inferior frontal gyrus, and orbitofrontal cortex) (25). Thus, olfactory impairment in AE patients could arise from functional disturbances to olfaction-related regions of the limbic system or from autoantibody-mediated increase and decrease of synaptic excitation or inhibition (3, 26). Previous neuropathological and radiological studies revealed a vulnerability of the hippocampus and amygdala to structural damage in patients with AE (5, 27–30). In this context, future MRI-studies using specific sequences such as diffusion tensor imagine (DTI) to detect possible structural damage of the olfactory pathway and the limbic system and correlate it with the olfactory capacity of AE patients would be of interest. Furthermore, it would be interesting to measure olfactory bulb (OB) and olfactory brain volumes of AE patients as these parameters seem to reflect well the olfactory capacity (31, 32). Acute inflammation in olfaction related regions of the limbic system could possibly lead to reduced olfactory bulb and olfactory brain volumes. The capability of neurogenesis in the subgranular zone of the hippocampus and subventricular zone of the lateral ventriceles was investigated in former studies (33–35). The migration of neuroblasts along the rostral migration stream to the OB leads to a high placticity of the OB. Thus, OF in AE patients could possibly improve after immunomodulatory treatment in the course of disease due to regeneration of OB.

The loss of peripheral olfactory function as well as of central olfactory function detected in our study might be explained by the cortical olfactory feedback system (35, 36). Aqrabawi et al. (37) identified the ventral hippocampus as a limbic input in a top-down modulation of olfactory sensitivity via pars medialis of the anterior olfactory nucleus to the olfactory bulb.

Olfactory dysfunction is not specific for AE patients. However, the association of olfactory dysfunction with neuroimmunological diseases was shown in different studies (38). In a recent MRI study the OF of multiple sclerosis and neuromyelitis optica spectrum disorder was compared (39). It was shown that the neuroanatomical features related to olfactory deficits differ between the two diseases, i.e., the same symptom is based on different pathomechanisms. To distinguish OF in AE patients from other neuroimmunological diseases further MRI studies comparing olfaction related brain regions of different disease entities would be of interest. In a recent prospective study, Armangue et al. (40) showed that 27% of HSE patients develop AE. In our study none of the 32 AE patients had a HSE in their medical history. An immunohistological study showed that HSV was present in the olfactory cortex and in glia cells of olfactory tracts (41). OF in HSE patients was hitherto not examined. In this context, it would be of interest to examine OF in HSE and to compare it with AE patients.

In detail, 26% of AE patients showed hypogeusia in gustatory testing and 80% of these patients also displayed olfactory impairment. Intersections between the olfactory and gustatory system on a cortical level might explain the high incidence of combined OD and GD due to damage in regions of the central nervous system like the amygdala, the orbitofrontal cortex, the thalamus or the insula (42–44).

In our study the OF and GF of AE patients was neither correlated with the presence or categorization of antibodies nor the disease duration most likely due to the small sample size of the subgroups. To analyze the impact of antibody status and antibody titer on OF and GF, four groups (antibodies against synaptic receptors, against intracellular antigens, against ion channels and other cell-surface proteins and without antibodies) ranging from 6 to 12 patients were formed (3).

A limitation of our study is the single-center design and a heterogeneous and small sample with few patients in the different antibody subgroups, related to the rarity of AE (45). Further studies with a larger number of patients with different AE syndromes are required to detect possible differences between different AE subgroups. That might further explain different pathomechanisms leading to olfactory and gustatory impairment in AE patients. Another limitation of our study is that patients with severe cognitive deficits and high physical disability in particular at an intensive care unit setting had to be excluded in order to participate at the different tests (46). Especially these patients might show increased structural damage to the limbic system possibly leading to more severe damage of the olfactory pathway. Thus, OF and GF might be even more severely impaired in AE patients than reflected in our results. Moreover, it is a limitation of our study that the effect of different immunomodulatory treatments on OF and GF was not examined. It would be interesting to investigate in a longitudinal study whether the severity of olfactory and gustatory dysfunction is depending on the immunomodulatory therapy.

In summary, this is the first study investigating olfactory and gustatory function in AE patients. We conclude that olfactory and gustatory dysfunction are hitherto uncharacterized symptoms in AE patients, similar to visual dysfunction in patients with NMDA receptor encephalitis (47). We suggest performing further studies with a larger number of patients. The Identification subtest could be used as an olfactory screening test in clinical routine in patients with suspected AE, as it is easy to perform, time and cost effective, and showed a high sensitivity and specificity to differentiate between AE patients and HC. It would be interesting to investigate in a longitudinal study whether changes in olfactory capacity might reflect treatment response to immunomodulatory therapy. Furthermore, the AE patients in our study were tested in average 19 months after disease onset. Both the examination of OF and GF in AE patients with acute disease onset and the longitudinal follow up testing are needed to evaluate the potential diagnostic and prognostic role of olfactory and gustatory testing in AE patients. In addition, our findings might contribute to a better understanding of disease pathomechanisms. Inflammation in olfactory and gustatory structures caused by autoantibodies in AE patients could explain the presence of olfactory and gustatory disturbances in these patients.

## Ethics Statement

Ethical approval was obtained by the Ethics Committee of Charité-University Hospitals Berlin. The study was conducted in accordance to the Declaration of Helsinki in its currently applicable version and applicable German laws. Written informed consent was approved by all participants.

## Author Contributions

RG: acquisition and interpretation of data, drafting of the manuscript and figures, statistical analysis, and interpretation of data. FU, KH, and KR: analysis of data and critical revision of manuscript for intellectual content. HP: acquisition of data, analysis of data, and critical revision of manuscript for intellectual content. FP: analysis of data, critical revision of manuscript for intellectual content, and obtaining funding. LH: study concept and design, analysis of data, and critical revision of manuscript for intellectual content. FS: study concept and design, study supervision, analysis of data, and critical revision of manuscript for intellectual content.

### Conflict of Interest Statement

The authors declare that the research was conducted in the absence of any commercial or financial relationships that could be construed as a potential conflict of interest.
